# Effects of Zusanli and Ashi Acupoint Electroacupuncture on Repair of Skeletal Muscle and Neuromuscular Junction in a Rabbit Gastrocnemius Contusion Model

**DOI:** 10.1155/2016/7074563

**Published:** 2016-04-13

**Authors:** Zhan-ge Yu, Rong-guo Wang, Cheng Xiao, Jun-yun Zhao, Qian Shen, Shou-yao Liu, Qian-wei Xu, Qing-xi Zhang, Yun-ting Wang

**Affiliations:** ^1^Department of Graduate School, Beijing University of Chinese Medicine, Beijing 100029, China; ^2^College of Acupuncture-Moxibustion and Tuina, Beijing University of Chinese Medicine, Beijing 100029, China; ^3^Institute of Clinical Medicine, China-Japan Friendship Hospital, Beijing 100029, China; ^4^School of Basic Medical Science, Beijing University of Chinese Medicine, Beijing 100029, China; ^5^Department of Tuina, Dongfang Hospital, Beijing University of Chinese Medicine, Beijing 100078, China; ^6^Department of Traditional Chinese Medicine Surgery, China-Japan Friendship Hospital, Beijing 100029, China; ^7^Department of Traditional Chinese Medicine, Northern Hospital, Beijing 100029, China; ^8^Department of Graduate School, Peking University of Health Science Center, Beijing 100029, China; ^9^Department of Orthopedics, China-Japan Friendship Hospital, Beijing 100029, China

## Abstract

*Objective*. To explore the effects of electroacupuncture (EA) at ST36 (EA-ST36) and at Ashi acupoints (EA-Ashi) on skeletal muscle repair.* Methods*. Seventy-five rabbits were randomly divided into five groups: normal, contusion, EA-Ashi, EA-ST36, and EA at Ashi acupoints and ST36 (EA-AS). EA (0.4 mA, 2 Hz, 15 min) was applied after an acute gastrocnemius contusion. The morphology of myofibers and neuromuscular junctions (NMJs) and expressions of growth differentiation factor-8 (GDF-8), acetylcholinesterase (AChE), Neuregulin 1 (NGR1), and muscle-specific kinase (MuSK) were assessed 7, 14, and 28 days after contusion.* Results*. Compared with that in contusion group, there was an increase in the following respective parameters in treatment groups: the number and diameter of myofibers, the mean staining area, and continuities of NMJs. A comparison of EA-Ashi and EA-ST36 groups indicated that average myofiber diameter, mean staining area of NMJs, and expressions of AChE and NRG1 were higher in EA-Ashi group, whereas expression of GDF-8 decreased on day 7. However, increases in myofiber numbers, expressions of MuSK and AChE, as well as decreases in GDF-8 expression, and the discontinuities were observed in EA-ST36 group on the 28th day.* Conclusion*. Both EA-ST36 and EA-Ashi promoted myofiber regeneration and restoration of NMJs. EA-Ashi was more effective at earlier stages, whereas EA-ST36 played a more important role at later stages.

## 1. Introduction

A contusion is a common sports injury and involves approximately 12.1% of all skeletal muscles disorders [1]. Contusions considerably impact people's lives and productivity [2]. The current treatment for acute skeletal muscle injury typically leads to secondary injury due to scar tissue formation [3]. In recent years, researches have focused on treating skeletal muscle injuries with growth factors, genetic engineering, and stem cell therapy. However, they have side effects and their clinical application is controversial [4]. Based on the meridian theory of traditional Chinese medicine (TCM), electroacupuncture (EA) plays a therapeutic role in conjunction with trace current wave stimulation to acupoints. Due to the curative effects of EA for skeletal muscle injuries [5], there is an increasing amount of research on skeletal muscle regeneration via EA [6].

Our preliminary study confirmed that EA at both Ashi acupoints and Zusanli (ST36) acupoint (EA-AS) could promote the regeneration of muscle [7] and improve the electrophysiological properties of the rabbit gastrocnemius (GM) after contusion [8]. However, the respective roles and mechanisms of EA at Ashi acupoint (EA-Ashi) and ST36 (EA-ST36) in skeletal muscle repair remain unclear.

In order to understand more details of the treatment and provide a laboratory basis for making better clinical decisions, we designed the present study to (1) determine whether EA-ST36 or EA-Ashi could improve skeletal muscle and neuromuscular junctions (NMJs) repair and (2) investigate the individual characteristics of EA-ST36 treatment and EA-Ashi treatment in the repair in a rabbit model with a GM contusion. To attain these goals, we evaluated the morphology of myofibers and NMJs. Furthermore, the mechanisms of the treatments were assessed by the following parameters: the expression of growth differentiation factor-8 (GDF-8) for muscle fiber regeneration, acetylcholinesterase (AChE) content, muscle-specific kinase (MuSK), and Neuregulin 1 (NGR1) for NMJ restoration.

## 2. Materials and Methods

### 2.1. Animal Models, Experimental Groups, and Treatment

Seventy-five New Zealand white rabbits (male or female, 2.0 ± 0.5 kg) were randomly divided into five groups: the normal group (*n* = 15), contusion group (*n* = 15), EA-Ashi group (*n* = 15), EA-ST36 group (*n* = 15), and EA-AS group (*n* = 15). All animals had access to food and water ad libitum in temperature (23 ± 1°C) and humidity (50 ± 5%) controlled rooms with 12-hour light-dark cycles. The experimental procedures were approved by the Ethical Committee of the Academy of Medical Sciences and were conducted in accordance with internationally accepted principles for laboratory animal use and care.

The rabbits were positioned with their right sides fixed to the experimental table after anesthesia with 3% pentobarbital sodium (30 mg/kg of body weight) through the marginal vein of the ear. The hindlimb was set by extending the knee and dorsiflexing the ankle to 90° to fully display the GM. The crushing machine was used to create a contusion with the drop-mass technique using 9.555 J of energy. The injured area was approximately 1 cm^2^ on the muscle bellies of the GM. The skin was confirmed to be intact, and there were no fractures.

**Figure 1:**
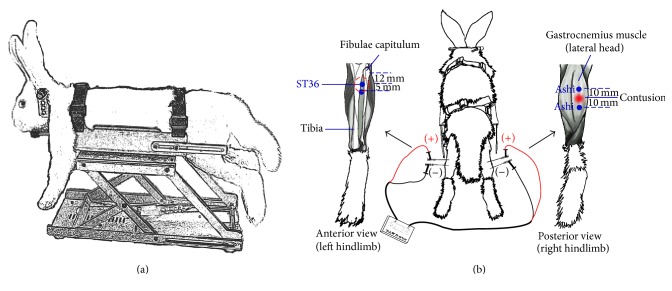
Outline of rabbit restraint and electroacupuncture (EA) stimulation. A specially designed fixator was applied to restrain the rabbits undergoing EA (a). A schematic diagram showing EA stimulation at ST36 and Ashi acupoints (b). ST 36 is located between the tibia and fibula, approximately 12 mm beneath the fibulae capitulum. Acupuncture needles were inserted perpendicularly as deep as 15 mm at left ST36 (anode) and 5 mm away from that (cathode) and kept in place. Another pair of acupuncture needles were inserted into the Ashi acupoints of right hindlimb at a depth of 15 mm, which were located 10 mm from the proximal (anode) and distal (cathode) end of the contusion midpoint. Each of the paired needles was then subjected to a current (0.4 mA, 2 Hz, 15 min) by Han's acupoint nerve stimulator.

To keep the injury site dry and avoid infection, 25 g/L anerdian was applied topically once per day. The animals were treated with EA 24 hours after contusion. In the EA-ST36 group, the main needle (anode (diameter: 0.25 mm, length: 25 mm), Zhongyan Taihe Medical Instruments Co. Ltd., Beijing, China) was inserted into ST36 (according to World Health Organization standards) [9] of the normal hindlimb with a depth of 15 mm. Then, the auxiliary needle (cathode) was placed 5 mm away from the main needle. On the injured hindlimb of rabbit in the EA-Ashi group, Ashi acupoints were located 10 mm from the proximal end (as the anode) and distal end (as the cathode) of the contusion midpoint. The acupoints in EA-AS group selected ST36 in the normal hindlimb and Ashi acupoints of the injured hindlimb of rabbit. When the needles were placed, all of the electrodes were stimulated synchronously with identical parameters (0.4 mA, 2 Hz, 15 min) using Han's acupoint nerve stimulator (Han's 200E, Nanjing Jisheng Medical Co. Ltd., Jiangsu, China) (Figure 1(b)). The rabbits in the contusion group and normal group received mock EA treatments, with the fixed position and time in the treatment groups but without EA treatments [10] (Figure 1(a)).

### 2.2. Material Collection

Five rabbits from each group were sacrificed by the air embolism method randomly on the 7th, 14th, and 28th day after contusion. The injured area of the GM was cut vertically into four equal parts after being washed by prechilled PBS. The first tissue (approximately 1.5 × 1.0 × 0.5 cm) was fixed in 4% paraformaldehyde for 24 hours. Then, the specimen was placed in 15% or 30% sucrose solution (mass fraction) until it sank down to the bottom of the container. After being embedded by optimum cutting temperature compound (4583, SAKURA, CA, USA), the samples were cut into 30 *μ*m sections parallel to the direction of the myofibers. The sections were stored at 4°C after drying at room temperature for immunofluorescence staining. Another set of samples was fixed in 4% formalin for 3 days and then processed via gradient alcohol dehydration and paraffin embedding. Then cut into 5 *μ*m sections, alcohol dewaxing and hydration were performed for hematoxylin-eosin (HE) and immunohistochemical staining. The remaining tissue portions were wrapped in foil, frozen in liquid nitrogen, and stored at −80°C for enzyme-linked immunosorbent assay (ELISA) and quantitative real-time polymerase chain reaction (QRT-PCR).

### 2.3. HE Staining of the Myofiber Morphology

After hydrating the tissue, the slides were dipped into a Coplin jar containing Mayer's hematoxylin and agitate for 30 sec. The slides were stained with 1% eosin Y solution for 10–30 sec via agitation and then rinsed in H_2_O for 1 min. The sections were then dehydrated with two applications of 95% alcohol and two applications of 100% alcohol for 30 sec each. The alcohol was then extracted with two applications of xylene. One drop of mounting medium was added, and the samples were covered with a coverslip. The slides were recorded and analyzed by microscopy (Zeiss Scope. AI, Carl Zeiss, Germany) with a 20x objective lens. Five random fields of each section were chosen for the analysis of morphological changes of injured skeletal muscles. The number of muscle fibers was recorded while the average diameter was measured in the obtained pictures using Image-Pro Plus Image (IPP, Version 6.0, Media Cybernetics, USA).

### 2.4. Immunohistochemical Staining of GDF-8

Tissue sections were placed in 3% H_2_O_2_ at room temperature for 10 min, rinsed with distilled water, and immersed in confining liquid for sealing. For the first antibody incubation, GDF-8 (1 : 50) rabbit polyclonal antibody (Santa Cruz, CA, USA) was incubated overnight at 4°C. After rinsing with distilled water, the second antibody, horseradish peroxidase- (HRP-) conjugated anti-rabbit IgG (Wuhan Boster Biological Engineering Co. Ltd., China), was incubated at 37°C for 30 min. After rinsing, histochemical stain 3,3′-diaminobenzidine (DAB, Wuhan Boster Biological Engineering Co. Ltd., China) was applied for visualization at room temperature for 10 min. After recording, five fields of each section were chosen, and the mean optical density (MOD) was computed using IPP.

### 2.5. Assessment of NMJ Morphology

**Figure 2:**
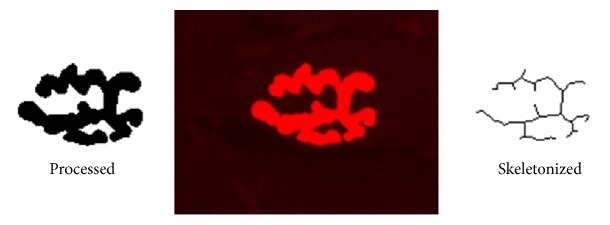
Processed and skeletonized images of neuromuscular junction (NMJ). Confocal photomicrograph of the NMJ (middle) was obtained with an OLYMPUS FV1000 confocal laser-scanning microscope. The staining area of NMJ was measured from processed binary images (left) and discontinuity was quantified using pixel positions of skeletonized images (right) generated in Image J (NIH) software.

A typical NMJ results from several twisting branches of the motor neurons. Each branch is enlarged to form the terminal synaptic boutons, which contain synaptic vesicles full of the acetylcholine (ACh). Boutons are located over stabilizing invaginations called junctional folds, where high-density clusters of acetylcholine receptors (AChRs) reside [11]. *α*-bungarotoxin (*α*-BTX) can bind to AChR. With help of fluorescent dye labeling, the morphological characteristics of NMJs can be observed and measured visually through fluorescent microscopy. The sections were washed with PBS fluid, and, then, marker profiles were obtained using Pap Pen, stained with *α*-BTX conjugated to rhodamine (T0195, Sigma, USA) (1 : 100, diluted by PBS) and protected from light for 1 hour. The sections were mounted with fluorescence-free glycerol after being washed with PBS fluid. Digital images of the NMJs were obtained with an OLYMPUS FV1000 confocal laser-scanning microscope with an excitation of 480 nm (40x objective for viewing and 60x objective for representative images). Five random NMJs were captured for each section; the maximum intensity flat plane projection was made from Z-stacked images in Image J software (NIH). Only NMJs in a complete* en face* view were selected for analysis. A Gaussian Blur filter with *σ* = 2.00 was applied after background was subtracted and noise despeckled. Mean stained area was quantified from binary images (Figure 2, processed). The connectivity of NMJs was described from skeletonized images (Figure 2, skeletonized) for each pixel via the number of neighbouring pixels. One neighbour implied a terminal pixel, two neighbours implied a pixel along a single branch, and three or more neighbours indicated that a pixel exists at a branch node. The terminal pixels were counted within the NMJ to describe discontinuities [12].

### 2.6. ELISA of AChE

The protein levels of AChE in the samples were quantified using ELISA. Homogenized tissues blending with equivalent 0.05 mol/L PBS (PH = 8) were coated to microtiter ELISA plates and incubated at 4°C overnight. After washing, the plates were blocked by a 1 h room temperature with 3% BSA. The AChE antibody (ab2803, Abcam Co. Ltd., UK) (0.2 mg/mL) was added to the plates, which were then incubated for 1 h at 37°C. HRP-conjugated anti-rabbit IgG was added to quantify the binding of the secondary antibody. After chromogenic assay by 0.03% o-phenylenediamine and stopping the reaction by the addition of 2 mol/L sulphuric acid, the optical density (OD) was measured on a microplate reader (DNM-9602, Perlong, Beijing, China) at 492 nm. This experiment was carried out twice on different dates under uniform laboratory conditions to avoid internal variations and thus to determine the reproducibility of the assay.

### 2.7. QRT-PCR for MuSK and NRG1

We used QRT-PCR to evaluate the RNA according to the fluorescence value of the amplified exponential phase. Samples were homogenized in Trizol reagent (15596-018, Invitrogen, USA), and total RNA was extracted according to the manufacturer's instructions. Subsequently, RNA was reverse transcribed (Revertaid First Strand cDNa Synthesis Kit, Thermo Scientific K1622, USA), and qRT-PCR was carried out with an ABI 7900HT Sequence Detection System (Applied Biosystems, USA) using SYBR green (SYBR FAST qPCR Kit Master Mix (2x) Universal, KAPA Biosystems, USA). Relative expression was determined by simultaneous comparison to the “housekeeping” gene, actin, using the geNorm software (v3.5, Ghent, Belgium). Transcriptions for MuSK and NGR1 were assessed. The primer sets used for PCR amplification were as follows: Actin-F1, CACACTCCCGCTCAGCTCAC and Actin-R1 GCTTGCTCTGGGCCTCGT; NGR1-F, CTTCGCTGTGAGACCAGTTCAG and NGR1-R CCAGTGATGCTTTGTTGATGC; Musk-F TGTTCTCCTGCCTGAGCCTG and Musk-R TTGCGGGTAGGATTCCACTG.

### 2.8. Statistics

The results were presented as the mean ± standard deviation. The data from each time point were analyzed with the SPSS 20.0 statistical software (SPSS Inc., Chicago, IL, USA). The normal distribution was analyzed using single factor analysis of variance. The groups were compared using the LSD method. The outcomes were evaluated using double-sided inspection.* P* < 0.05 was considered to be statistically significant.

## 3. Result

### 3.1. Evaluation of Muscle Regeneration after Contusion

**Figure 3:**
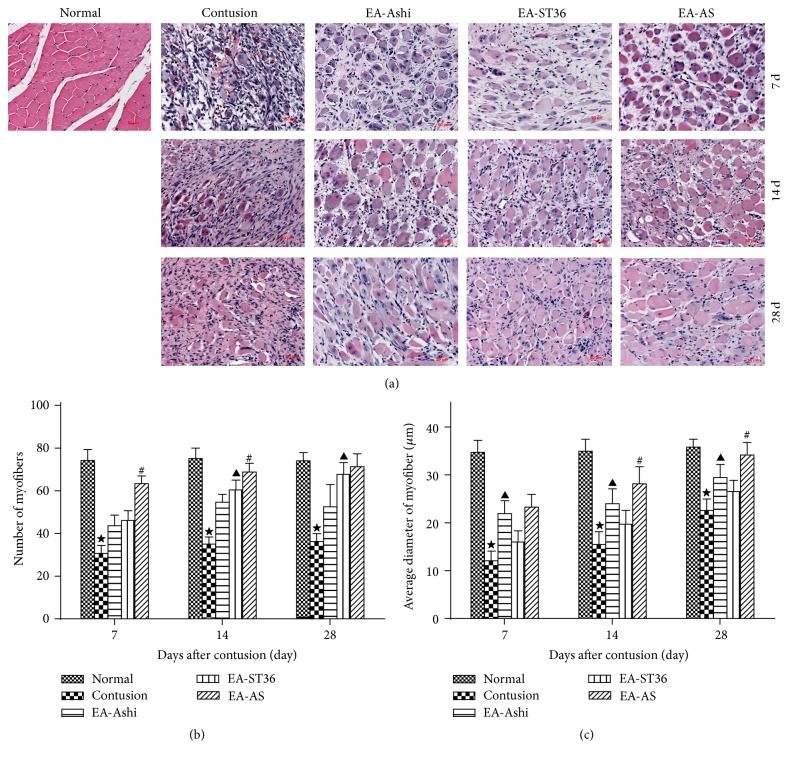
Tissue sections were stained with hematoxylin and eosin (HE) to evaluate the general morphology of muscle fibers. HE staining (×200) of normal skeletal muscle revealed fibers uniform in size containing peripherally located nuclei. After contusion, there was skeletal muscle degeneration and necrosis characterized by variation in the size and shape of skeletal muscle fibers, granular and/or hyalinized eosinophilic cytoplasm. Enhanced mononuclear cell infiltration was detected in the connective tissue between muscle fibers. Over time, regenerating myofibers that are centrally nucleated and heterogeneous in size could be detected. By day 28 after contusion, some regenerating fibers were mature, especially in the group receiving EA-AS, peripherally nucleated, and homogeneous in size (a). Quantitative analyses of the number (b) and average diameter (c) of skeletal muscle fibers were administrated on the 7th, 14th, and 28th day after contusion. Compared to the untreated group (contusion), the treatment groups (EA-Ashi, EA-ST36, and EA-AS) showed an increase in number and average diameter of myofibers (*P* < 0.05). EA-ST36 increased more myofiber number especially from two weeks (*P* < 0.05) and EA-Ashi was more effective on enhancing average diameter of myofibers (*P* < 0.05) by comparison between them (EA-Ashi versus EA-ST36, ^▲^ *P* < 0.05; EA-AS versus EA-Ashi and EA-ST36, ^#^ *P* < 0.05; contusion group versus treatment groups, ^★^ *P* < 0.05; EA-Ashi, electroacupuncture at Ashi acupoints; EA-ST36, electroacupuncture at ST36; EA-AS, electroacupuncture at Ashi acupoints and ST36; treatment groups (EA-Ashi, EA-ST36, and EA-AS)).

During muscle regeneration, new skeletal muscle fibers exhibited their central nuclei and then matured into periphery nucleated fibers. The regenerative myofiber cytoplasm and large hyperchromatic nuclei were identified in the center of cell using basophilic blue staining [13]. The HE staining results showed that the diameter and volume of the myofibers increased gradually and that the nucleus deviated toward the periphery during the repair process (Figure 3).

### 3.2. Expression of GDF-8 in Each Group

**Figure 4:**
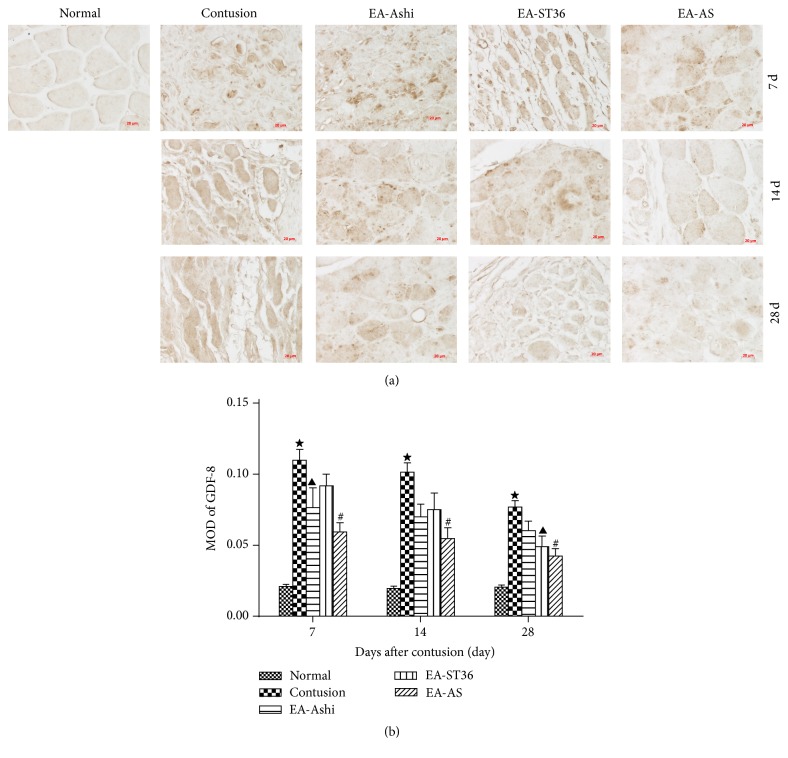
Immunohistochemical staining of growth differentiation factor-8 (GDF-8) in the skeletal muscle. Muscle cytoplasm reacting with GDF-8 was stained brown. The staining of GDF-8 was very weak in the normal muscle tissue. But, after contusion, muscle fibers showed strong immunoreaction for GDF-8 (a, ×400). The expression peaked on the 7th and 14th day and decreased significantly by the 28th day. The mean optical density (MOD) was assessed to quantify expression of GDF-8 in different groups. GDF-8 expression was higher in the contusion group compared to the treatment groups (EA-ST36, EA-Ashi, and EA-AS groups) (*P* < 0.05). Compared with the EA-ST36 group, GDF-8 expression in the EA-Ashi group was lower (*P* < 0.05) on day 7 but became higher on the 28th day (*P* < 0.05) (b) (EA-Ashi versus EA-ST36, ^▲^ *P* < 0.05; EA-AS versus EA-Ashi and EA-ST36, ^#^ *P* < 0.05; contusion group versus treatment groups, ^★^ *P* < 0.05; EA-Ashi, electroacupuncture at Ashi acupoints; EA-ST36, electroacupuncture at ST36; EA-AS, electroacupuncture at Ashi acupoints and ST36; treatment groups (EA-Ashi, EA-ST36, and EA-AS)).

GDF-8 was a muscle-specific factor in the TGF-*β* super family and an important myostatin that not only inhibits the regeneration of myofibers but also stimulates the differentiation of myofibroblasts [14]. Positive immunohistochemical staining of GDF-8 was observed in the cytoplasm of myofibers and fibrocytes (Figure 4).

### 3.3. Changes in NMJ Morphology in Each Group

**Figure 5:**
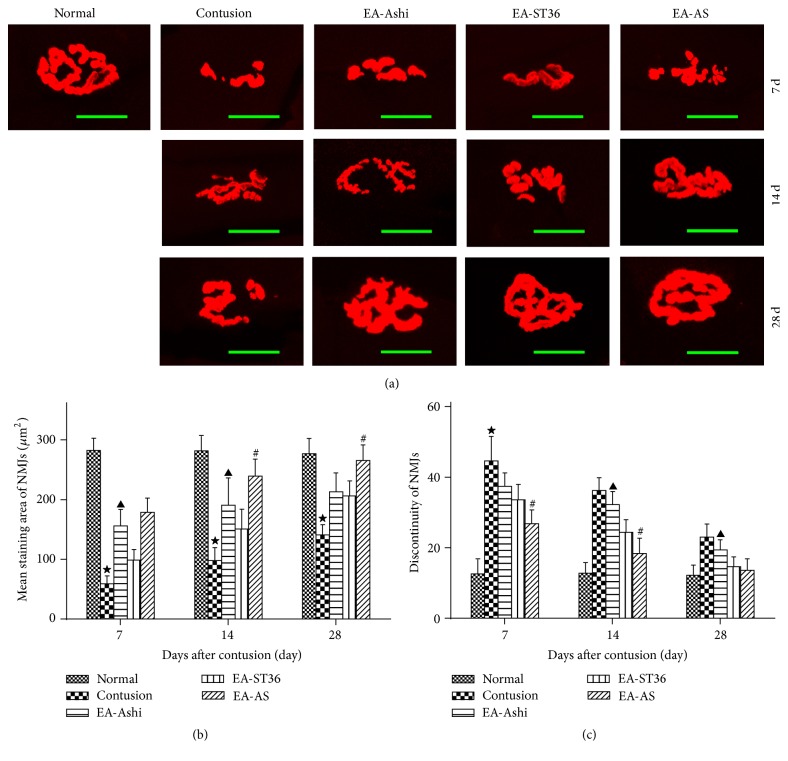
Morphology of neuromuscular junctions (NMJs) at different time points after contusion. Postsynaptic NMJ was identified by rhodamine *α*-bungarotoxin staining for acetylcholine receptors (AChRs) and observed by confocal microscopy. Representative confocal photomicrographs of NMJ (scale scar: 20 *μ*m) displayed the differences among all groups following contusion (a). In normal muscle fibers, AChR clusters were organized in a classical pretzel-shaped structure. NMJs showed a frequent fragmented appearance on day 7 after contusion: AChR staining in muscles was generally dramatically disorganized, with its branches barely discernable. On the 14th and 28th day, NMJs appeared to have some branches and be less fragmented, and the size of the postsynaptic apparatus had increased. Quantitative analysis of NMJs revealed that the mean stained area (b) increased and discontinuities (c) of NMJs decreased gradually during repair. The treatment (EA-Ashi, EA-ST36, and EA-AS) increased mean stained area and reduced discontinuity of NMJs compared to contusion. EA-ST36 decreased more discontinuity (*P* < 0.05), especially at the second and third week, and EA-Ashi was more effective during the first two weeks on enhancing mean stained area of NMJs, by comparison (*P* < 0.05) (EA-Ashi versus EA-ST36, ^▲^ *P* < 0.05; EA-AS versus EA-Ashi and EA-ST36, ^#^ *P* < 0.05; contusion group versus treatment groups, ^★^ *P* < 0.05; EA-Ashi, electroacupuncture at Ashi acupoints; EA-ST36, electroacupuncture at ST36; EA-AS, electroacupuncture at Ashi acupoints and ST36; treatment groups (EA-Ashi, EA-ST36, and EA-AS)).

Sections were stained with *α*-BTX for quantification and visualization. NMJs contained many wrinkles and appear to be plump and large “rosette-shaped” under normal conditions. In the samples, the NMJs degenerated and fragmented into oval-shaped patches one week after contusion, resulting in many dispersed synapses. With AChR aggregation, some of the synapses connected to one another, similar to short rods, and became larger in size a week later. NMJs in the contusion area exhibited a higher number of folds and were better stacked on the 28th day (Figure 5(a)). In addition, EA at different acupoints caused the mean stained area and discontinuities of the NMJs to change, as shown in Figures 5(b) and 5(c), respectively.

### 3.4. AChE Expression in the Muscles

**Figure 6:**
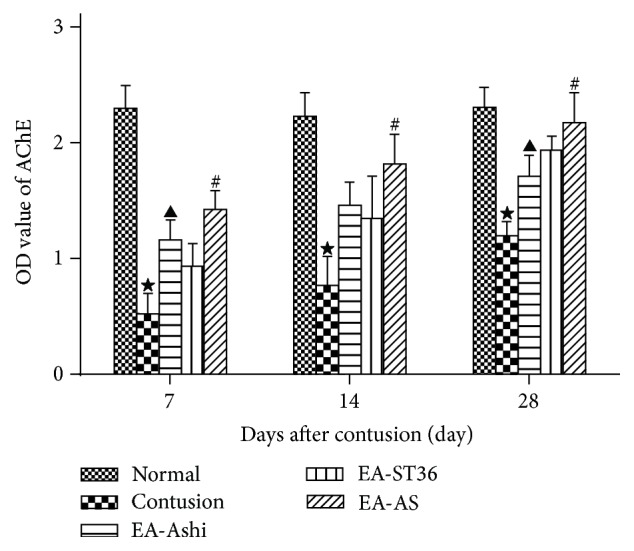
Content of acetylcholinesterase (AChE) in the recruited muscles. The optical density (OD) value at 492 nm obtained by ELISA was provided for skeletal muscle samples for the AChE protein. Increases in AChE content were observed in the treatment groups compared to that of contusion group at different time points during repair (*P* < 0.05). Compared with that of the EA-ST36 group, AChE content in the EA-Ashi group was higher on day 7 but became lower on the 28th day (*P* < 0.05) (EA-Ashi versus EA-ST36, ^▲^ *P* < 0.05; EA-AS versus EA-Ashi and EA-ST36), ^#^ *P* < 0.05; contusion group versus treatment groups, ^★^ *P* < 0.05; EA-Ashi, electroacupuncture at Ashi acupoints; EA-ST36, electroacupuncture at ST36; EA-AS, electroacupuncture at Ashi acupoints and ST36; treatment groups (EA-Ashi, EA-ST36, and EA-AS)).

AChE is a pivotal enzyme in cholinergic nerve conduction and an important marker for the function of cholinergic neurons [15]. The protein levels of AChE were assessed by ELISA. The results showed that AChE content was considerably reduced after 7 days in the contusion group compared with that of the normal group. The content increased gradually in the following 3 weeks and approached normal by the 28th day (Figure 6).

### 3.5. MuSK mRNA and NRG1 mRNA Expressions in the Muscles

**Figure 7:**
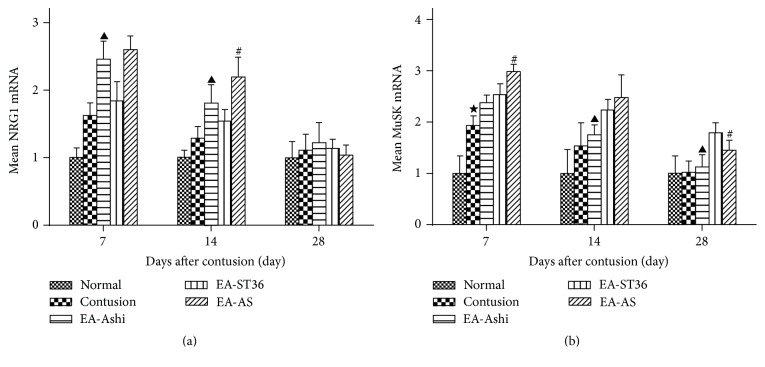
Comparison of the expression of Neuregulin 1 (NGR1) and muscle-specific kinase (MuSK) at different time points after contusion. QRT-PCR assays were conducted with samples from rabbits to demonstrate the gene expressions of NRG1 (a) and MuSK (b). Both MuSK and NRG1 expressions were increased on the 7th day after contusion and gradually decreased near to normal in the following 3 weeks. Comparison between EA-Ashi and EA-ST36 groups showed that mean NRG1 mRNA in EA-Ashi group was higher than that of EA-ST36 group on day 7 and day 14 after contusion (*P* < 0.05), while mean MuSK mRNA in the EA-ST36 group was lower on the 14th and 28th day (*P* < 0.05) (EA-Ashi versus EA-ST36, ^▲^ *P* < 0.05; EA-AS versus EA-Ashi and EA-ST36, ^#^ *P* < 0.05; contusion group versus treatment groups, ^★^ *P* < 0.05; EA-Ashi, electroacupuncture at Ashi acupoints; EA-ST36, electroacupuncture at ST36; EA-AS, electroacupuncture at Ashi acupoints and ST36; treatment groups (EA-Ashi, EA-ST36, and EA-AS)).

NMJs occur in a specialized area of the sarcolemma called the motor end-plate (MEP). Reconstruction of NMJ function requires a large number of AChRs aggregated in the MEP. This process is mainly regulated by the Agrin/MuSK pathway [16]. MuSK is a transmembrane protein tyrosine kinase (PTK) that is expressed and aggregated in the postsynaptic membrane coupled with AChR, which is more important for AChR clusterization regulation than Agrin [17]. Moreover, transcriptional regulation has a pivotal role in AChR clusterization. Recent evidence has indicated that the NRG-1/ErbB pathway is the main signal responsible for the increased transcription of AChR [18]. The expressions of key genes in the aforementioned pathways were tested by QRT-PCR, as presented in Figure 7.

## 4. Discussion

Clinically, EA is widely applied in skeletal muscle injury treatment [5], and research on the mechanism of EA has attracted increasing attention. TCM held that the basic pathological change after contusion was “qi-stagnation and blood stasis.” Therefore, “activating blood and regulating qi” were the key to treatment. According to the theory that “where there is pain, there is an acupoint” in “Miraculous Pivot,” the Ashi acupoint near the injured area is typically chosen to treat traumatic injury [19–21]. ST36 is he-sea acupoint on the stomach meridian of foot-yangming, and the yangming meridian is full of blood and qi in meridian theory. Therefore, acupuncture at ST36 could regulate the overall qi and blood circulation and strengthen the healthy qi of the body. Modern research has also demonstrated that acupuncture at Ashi acupoint could activate blood and disperse blood stasis [22]. EA-ST36 could not only protect organisms from injury [23] but also promote general body recovery after injury [24, 25].

In injured skeletal muscle, it is generally accepted that the Ashi acupoint is the injured region. To avoid new injury generated from needles inserted into injured area, Ashi acupoints were not located in the exact contusion midpoint, but rather 10 mm [26] from the proximal and distal end of it in our treatment protocol. To regulate the overall qi (remote effect of ST36) and avoid interfering Ashi acupoints (near to one of the Ashi acupoints), ST36 of the normal hindlimb was selected. Because the distance between the two needles is 1 mm in rat, if there is only one acupoint located ipsilaterally, according to common use in previous studies [27, 28], we made the distance 5 mm in rabbit by a combination of comparative anatomy, simulated bone length measurement, and experimental observation. Earlier experiments confirmed that EA following this treatment protocol could improve the regeneration [7] and electrophysiological properties [8] of myofibers. However, it was unclear whether EA-ST36 and EA-Ashi could individually play a role in promoting the repair. Furthermore, the respective characteristics and mechanism of the effects of the EA-ST36 and EA-Ashi were unclear. This experiment explored the above topics by observing the effects of EA-ST36 and EA-Ashi acupoints on the morphology of skeletal muscle fibers and NMJs as well as the expressions of GDF-8, AChE, NRG1, and MuSK at different times over the course of repair.

### 4.1. By Inhibiting the Expressions of GDF-8, EA-ST36 and EA-Ashi Acupoints Could Increase the Number and Diameter of Myofibers. The Effect of EA-ST36 Was Delayed with respect to That of EA-Ashi, but the Long-Term Effect Was Better

After contusion, the number and diameter of muscle fibers in the treatment groups were significantly increased compared to the contusion group, indicating that both EA-ST36 and EA-Ashi could promote skeletal muscle regeneration. The diameter increased more significantly in the EA-Ashi group. This greater increase in diameter might be related to the fact that the electrodes were placed on the distal and proximal ends of the contusions, as muscle fibers might adaptively thicken against the mechanical stretching generated by electrodes [29, 30]. In addition, regional electrical stimulation promoted the growth of denervated muscles [10, 31] or indirectly inhibited the metabolism of myofibers so that they were relatively enlarged [32]. EA-ST36 was more effective in increasing the number of muscle fibers than EA-Ashi, likely because EA-ST36 was more effective in promoting cell proliferation [33] and angiogenesis [24]. The precise mechanisms of action need to be investigated in future research. EA-Ashi was more effective in reducing the expression of GDF-8 on the 7th day, whereas EA-ST36 was more effective two weeks later. The effect of EA-ST36 was delayed compared to that of EA-Ashi, but the long-term effect was better. A previous study found that regional EA at “Heyang and Feiyang” acupoints (amounting to Ashi acupoints in this study) could lower the expression of myostatin [24]. This research has found that EA-ST36 had a similar function and was more effective on inhibiting the expression of GDF-8 compared to EA-Ashi in the last two weeks.

### 4.2. Both EA-ST36 and EA-Ashi Promoted the Reconstruction of NMJ and EA-Ashi Could Increase the Expressions of AChR and AChE in the Earlier Stage, Whereas EA-ST36 Could Promote AChR Clusterization and the Expression of AChE at a Later Stage

The most important event in the restoration of a NMJ is the expression and aggregation of AChR on the MEP. In this study, stained *α*-BTX was used to measure the MEP of the rabbit GM at different time points after muscle damage by confocal laser-scanning microscopy. The mean staining area and discontinuities of the MEPs were calculated. The discontinuities reflected the clusterization of AChRs, whereas the mean staining area represents the volume of AChRs in each NMJ. The majority of NMJs in damage muscle had degraded, appearing as scattered oval-shaped plaques at 7 days after injury, indicating secondary damage to the NMJs after muscle contusion. As AChRs gradually gathered, the NMJ was partially reconstructed after 2 weeks and some cracks appeared. The scattered AChRs connected into sheets, and the NMJ became oval at 4 weeks after contusion, although many NMJs exhibited shrinking and dispersed shapes. Meanwhile, the mean stained area of the NMJs had become larger and the discontinuities had decreased. Aside from shape changes, we also observed that AChE increased gradually from days 7 to 28, indicating that the function of injured NMJs had been restored step by step. In contrast, the NMJs in the treatment groups exhibited fuller shapes with more folds, larger mean staining area, lower discontinuities, and a higher content of AChE, indicating that EA may promote the survival of NMJs and thus play a pertinent role in accelerating their recovery. Previous studies have shown that EA could promote the survival of injured neurons after transection of the neural stem [34] and that electrical stimulation accelerated NMJ formation [35]. These findings provide further support that EA improves the reconstruction of NMJ degeneration following skeletal muscle damage.

Furthermore, increases in the mean stained area of the NMJs and AChE expression were observed in the EA-Ashi and EA-AS groups on days 7 and 14, compared to that of the other groups. The treatments in these groups may potentially be better at increasing the proliferation of AChR in the early stage of repair. Although the mean stained area in EA-ST36 group was lower, the continuities and AChE content increased on 14 and 28 days after contusion, suggesting that the clusterization of AChR and NMJ's function had been enhanced.

We next explored the molecular mechanism of how this happens. As we know, AChR first proliferated after injury and then regressed following clusterization in the reconstruction and development processes of the NMJ [36]. NRG1 is the key factor responsible for the increased transcription of AChRs and MuSK regulates AChRs clusterization [37]. In this study, expressions of NRG1 and MuSK gradually decreased and remained higher than the normal level on day 14 after contusion, possibly because the injured muscle stayed in a relaxed state after the loss of control from motor neurons, such that the muscle nuclei lost their inhibition by muscle activity [38]. On day 28, the expressions had decreased significantly, whereas the mean staining area of the NMJs and AChE expression had increased, possibly due to the synapse regression to improve the efficiency of synaptic transmission [36]. What is more, increases in the expression of NRG1 were observed in the EA-Ashi and EA-AS groups on days 7 and 14, while the MuSK increased significantly in EA-ST36 and EA-AS groups at the 14th and 28th day, compared to the other groups. These results may illustrate that EA-Ashi was more effective in improving the proliferation of AChR through regulating NRG1 mainly at earlier stages, whereas EA-ST36 was more effective in promoting the clusterization of AChR by regulating MuSK at later stages. By applying ST36 and Ashi acupoints simultaneously, EA could proliferate and cluster AChR more effectively within an appropriate timeframe.

## 5. Conclusion

In conclusion, in this study, we demonstrate via morphological and molecular biological approaches that both EA-Ashi and EA-ST36 have an effect on the regeneration of myofibers and restoration of NMJ, leading to muscle repair, possibly through the regulation of GDF-8, AChE, NRG1, and MuSK. EA-Ashi was more effective at earlier stages, whereas EA-ST36 was more effective later on. The combination of these two acupoints achieved a better effect than either one. Therefore, EA-AS is a suggested method for skeletal muscle injury treatment, which combines regional and distal acupoints. Additional molecular investigations are needed to clarify whether it is more effective to increase the stimuli of EA-Ashi and decrease EA-ST36 stimuli at earlier stages and reverse later on than the same stimuli of EA-AS.
